# Non-Contrast-Enhanced MR Angiography at 3 Tesla in Patients with Advanced Peripheral Arterial Occlusive Disease

**DOI:** 10.1371/journal.pone.0091078

**Published:** 2014-03-07

**Authors:** Kolja M. Thierfelder, Georgios Meimarakis, Konstantin Nikolaou, Wieland H. Sommer, Peter Schmitt, Philipp M. Kazmierczak, Maximilian F. Reiser, Daniel Theisen

**Affiliations:** 1 Department of Clinical Radiology, Ludwig-Maximilians-University Hospital, Munich, Germany; 2 Department of Vascular and Endovascular Surgery, Ludwig-Maximilians-University Hospital, Munich, Germany; 3 Siemens AG, Healthcare Sector, Erlangen, Germany; Department of Medicine and Biomedical Sciences, University of Algarve, Portugal

## Abstract

**Purpose:**

The aim of this study was to assess the diagnostic performance of ECG-gated non-contrast-enhanced quiescent interval single-shot (QISS) magnetic resonance angiography at a magnetic field strength of 3 Tesla in patients with advanced peripheral arterial occlusive disease (PAOD).

**Method and Materials:**

A total of 21 consecutive patients with advanced PAOD (Fontaine stage IIb and higher) referred for peripheral magnetic resonance angiography (MRA) were included. Imaging was performed on a 3 T whole body MR. Image quality and stenosis diameter were evaluated in comparison to contrast-enhanced continuous table and TWIST MRA (CE-MRA) as standard of reference. QISS images were acquired with a thickness of 1.5 mm each (high-resolution QISS, HR-QISS). Two blinded readers rated the image quality and the degree of stenosis for both HR-QISS and CE-MRA in 26 predefined arterial vessel segments on 5-point Likert scales.

**Results:**

With CE-MRA as the reference standard, HR-QISS showed high sensitivity (94.1%), specificity (97.8%), positive (95.1%), and negative predictive value (97.2%) for the detection of significant (≥50%) stenosis. Interreader agreement for stenosis assessment of both HR-QISS and CE-MRA was excellent (κ-values of 0.951 and 0.962, respectively). As compared to CR-MRA, image quality of HR-QISS was significantly lower for the distal aorta, the femoral and iliac arteries (each with p<0.01), while no significant difference was found in the popliteal (p = 0.09) and lower leg arteries (p = 0.78).

**Conclusion:**

Non-enhanced ECG-gated HR-QISS performs very well in subjects with severe PAOD and is a good alternative for patients with a high risk of nephrogenic systemic fibrosis.

## Introduction

Peripheral arterial occlusive disease (PAOD) has a prevalence of 12 to 20% in individuals of over 65 years and is one of the most important and cost-intensive vascular diseases [1]. Cross-sectional diagnostic imaging can determine severity and localization of stenosis and is therefore indispensable for planning of surgical and interventional procedures.

CT angiography (CTA) offers a high spatial resolution without the risks of an invasive procedure [2]. However, well known drawbacks of contrast-enhanced CT are the risk of iodinated contrast nephropathy and the ionizing radiation. As an alternative, contrast-enhanced MR angiography (CE-MRA) employing gadolinium-based contrast agents has shown to be equally accurate for the detection of vascular stenosis within the entire lower limb without a disturbing overlay from bone or calcified plaques [3]. Thus, CE-MRA is used as the primary imaging modality for PAOD patients in many centers [4].

However, since the risk of nephrogenic systemic fibrosis (NSF) in patients with chronic renal failure was reported [5], [6], interest in non-enhanced MR angiography techniques has markedly increased [7]–[9]. This applies in particular to the diagnostic investigation of patients suffering from PAOD as this population has a high prevalence of chronic renal failure [10]. To compound this problem, a complete CE-MRA investigation from the distal aorta to the pedal arteries typically requires a relatively large amount of contrast agent.

In recent studies, the non-enhanced electrocardiogram (ECG)-triggered quiescent interval single shot (QISS)-MR angiography has shown promising results [11]–[16]. On 1.5 Tesla MRI systems, this two-dimensional sequence proved to be resistant against changes in heart beat and showed good accuracy in vessels with high grade stenosis. At a slice thickness of 3 mm, sensitivity ranged from 84.9% to 98.6% and specificity from 94.6% to 96.8% [11]–[13], [16].

Despite of the positive results, further research is needed before QISS-MRA can be employed for general clinical use in PAOD patients. Reported drawbacks include poor image quality in the distal aorta and iliac arteries as well as the tendency to overestimate stenosis [13]. In previous studies, subjects with suspected PAOD but no significant stenosis were part of the patient collective. The inclusion of patients with a good vascular status, however, can lead to an overestimation of the diagnostic accuracy of QISS as MR imaging in PAOD patients has shown to be more challenging. With respect to these points, a systematic evaluation of the QISS-MRA on state-of-the-art high field MR systems in patients with advanced PAOD seems worthwhile.

The aim of this study was to assess the diagnostic performance of a high-resolution prototype of QISS-MRA (HR-QISS) in comparison to the reference standard of CE-MRA in patients with known PAOD. In contrast to previous studies, HR-QISS was performed on a 3 Tesla system, allowing for a slice thickness of 1.5 mm for the whole scan volume at a reasonable imaging time.

## Materials and Methods

This prospective study was approved by the local ethics committee of the Medical Faculty of the Ludwig-Maximilians-Universtity Munich, Germany. Between April 2012 and March 2013, 21 consecutive patients with advanced peripheral arterial occlusive disease (PAOD) were included. All patients had been referred for MRA of the lower extremities. Written informed consent was obtained from all patients. We evaluated the diagnostic performance and image quality of the non-enhanced HR-QISS MRA in comparison to contrast-enhanced MRA as standard of reference.

Inclusion criteria were (1) known advanced PAOD (Fontaine Stage IIb to IV [17]), and (2) the clinical indication for peripheral cross-sectional angiography of the lower extremities. Exclusion criteria were (1) contraindications to MRI (cardiac pacemaker, cochlea implants etc.), (2) renal impairment as indicated by point-of-care testing of an estimated glomerular filtration rate of less than 30 ml/min/1.73 m^2^, (3) known adverse reactions to gadolinium chelates, and (4) the inability to complete non-enhanced HR-QISS and/or CE-MRA.

Imaging was performed on a 3 Tesla whole body MR scanner (MAGNETOM Verio, Siemens Healthcare, Erlangen, Germany), with the patients in supine position and with feet first. In order to cover all arteries from the distal aorta to the pedal vessels, we used a dedicated peripheral angiography array coil with a 36-element coil design, supplemented by one or two 6-element body matrix coils to cover pelvis and lower abdomen and 12 to 16 elements of the spine matrix coil depending on the patient’s size.

### Non-enhanced HR-QISS

**Figure 1:**
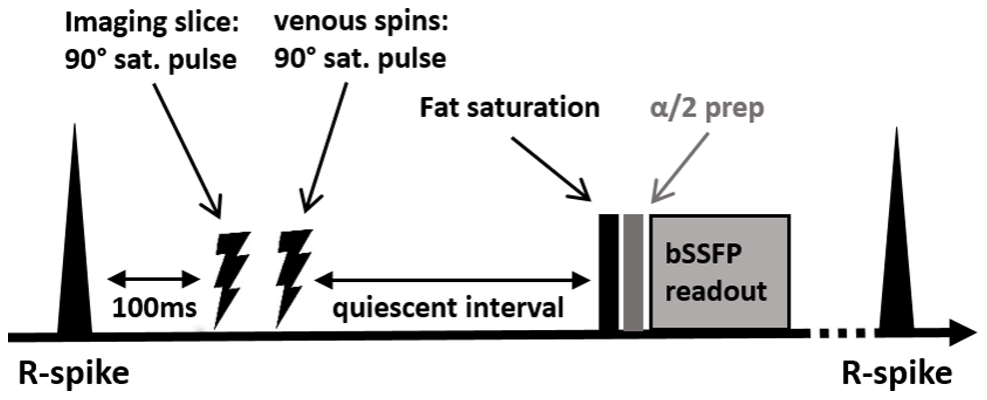
Pulse sequence diagram of the QISS sequence (modified after: Edelman RR et al., MRM 2009) [14]. Saturation of imaging slice and tracking saturation of venous signal are applied 100 ms after the R-spike. The subsequent quiescent interval allows for inflow of non-saturated blood spins before a single-shot 2D image is acquired.

QISS is an ECG-triggered single-shot two-dimensional balanced steady-state free precession (bSSFP) pulse sequence that was introduced in 2010 [14]. The quiescent interval as the key feature of the sequence is a waiting period after the R-spike without any radiofrequency excitation (Figure 1). The idea of the quiescent interval is that it coincides with the period of fast systolic arterial flow. By insuring maximum inflow of unsaturated spins into the investigated slice, a better contrast between the vessel and the surrounding soft-tissues is expected. Presaturation is used to suppress background tissue and venous signal while arterial signal is enhanced due to inflow into the investigated slice from proximal to distal during the quiescent interval. The signal acquisition is prepared by an alpha/2 preparation pulse, which is played out before the first excitation.

We used a non-commercial version of the QISS sequence that was modified for use in this research study. In contrast to previous studies, a slice thickness of only 1.5 mm and no overlap between adjacent slices was used for all regions from the distal aorta to the pedal arteries. In order to cover all vessels from the level of the infrarenal aorta to the pedal arteries, seven contiguous groups of 128 slices were acquired, covering 192 mm each. No breath holding maneuvers were used for any of the seven groups. Detailed information of the imaging parameters of HR-QISS and CE-MRA are presented in Table 1.

**Table 1:** Imaging parameters of HR-QISS and contrast enhanced MRA sequences.

Imaging Parameter	Non-enhanced HR-QISS	Contrast enhanced MRA	Contrast enhanced dynamic (TWIST)
Acquisition mode	2D	3D	3D
Acquisition time	896 cardiac cycles^1^	2∶24 min	1∶44 min
TR (msec)	3.35	2.81	3.03
TE (msec)	1.36	1.09	1.11
Flip angle (degrees)	50–60	24	24
Field of View (mm)	400×260×192	350×263×350	350×263×350
Voxel size (mm)	1.0×1.0×1.5	1.0×1.0×1.3	1.2×1.2×2.5
Phase partial Fourier factor	5/8	6/8	6/8
Slice partial Fourier factor	–	6/8	6/8
GRAPPA acceleration factor	Two	Three	Three
Bandwidth (Hz/px)	833	1000	650
Fat suppression	Yes	No	No

1 HR-QISS acquisition time depends on the subjects’ heart rate; GRAPPA: Generalized autocalibrating partially parallel acquisition; HR-QISS: High-resolution quiescent interval single shot sequence; MRA: Magnetic resonance angiography; TE: Echo time; TR: Repetition Time; TWIST: Time-resolved imaging with stochastic trajectories.

### Contrast-enhanced MRA (CE-MRA)

CE-MRA was performed with gadobutrol (Gadovist, Bayer Schering Pharma, Berlin, Germany) using a dual-injection technique consisting of a continuously moving table (CMT) MRA [18], followed by a time-resolved MRA for dedicated dynamic imaging of the calves [19].

First, a test bolus of 2 ml diluted gadobutrol (1 ml gadobutrol, 1 ml 0.9% NaCl) was administered at a flow rate of 1 ml/sec, its arrival was monitored using a dynamic 2D FLASH protocol in the abdomen. The signal in the abdominal aorta was used to determine the bolus arrival time. The subsequent CMT acquisition covered the whole arterial tree from the infrarenal aorta to the pedal arteries. It was obtained with an injection of 20 ml diluted gadobutrol (10 ml gadobutrol, 10 ml 0.9% NaCl) at a flow rate of 1 ml/sec, immediately followed by two 25 ml saline chasers at flow rates of 1 ml/sec and 0.5 ml/sec, respectively.

For the dynamic imaging of the arteries of the calves, a coronal time-resolved imaging sequence with stochastic trajectories (TWIST) [19] utilizing 10 ml diluted gadobutrol (5 ml gadobutrol, 5 ml 0.9% NaCl) at a flow rate of 1 ml/sec followed by a 25 ml saline chaser was employed.

### Image Analysis

All images were viewed on a 3D workstation using standard three-dimensional reconstruction software (syngoMMWP VE26A, Siemens Healthcare, Erlangen, Germany). Readings of HR-QISS and CE-MRA were performed separately by two blinded and board-certified radiologists with 12 (K.N.) and 10 years (D.T.) of experience in MRA, respectively. In case of disagreement between the readers, a consensus was reached in a separate session. Source images, multiplanar reformatted reconstructions (MPRs), and maximum intensity projections (MIPs) were allowed to be used for all datasets. Readers were blinded to the patients’ medical histories, clinical findings, and previous examinations.

The degree of stenosis and image quality were graded for 27 anatomic segments in each patient: for the infrarenal aorta and for 13 further segments in each lower extremity: common iliac artery, external iliac artery, common femoral artery, proximal half of superficial femoral artery, distal half of superficial femoral artery, popliteal artery, proximal half of anterior tibial artery, distal half of anterior tibial artery, tibioperoneal trunk, proximal half of peroneal artery, distal half of peroneal artery, proximal half of posterior tibial artery, and distal half of posterior tibial artery [13].

The degree of vessel stenosis was graded visually and in accordance with previous studies on non-enhanced QISS-MRA [12]–[14]. The degree of stenosis was defined in each vascular segment using a 5-point Likert scale: 0, normal; 1, luminal narrowing of <50%; 2, one lesion with luminal narrowing of ≥50%; 3, two or more lesions with luminal narrowing of ≥50%; 4, occlusion. Segments with grades from 2 to 4 were considered as significantly stenosed.

CE-MRA was represented by both CMT- and dynamic TWIST-MRA. In accordance to previous major trials, CMT-MRA was used for the arterial tree from the distal aorta to the popliteal arteries, while TWIST-MRA served as the reference standard for the calves [13], [20]. We did not include the TWIST-MRA as a third technique in our statistical analysis as it covered only the calves.

Image quality was rated for both CE-MRA and HR-QISS using a 5-point Likert scale: (0) non-diagnostic; (1) poor; (2) fair; (3) good; (4) excellent. This rating included the issue of venous contamination which is a common problem in MRA of the peripheral arteries. Segments with stents or arterial prosthesis were excluded from analysis due to artifacts in both techniques. Any other failure to visualize a segment using one of the techniques was considered an incorrect assessment by the respective sequence. For the evaluation of image quality, segments with no visible artery due to total occlusion in the whole segment of the evaluated artery were excluded.

### Statistical Analysis

We performed all statistical analyses using SPSS Statistics 20 (IBM, Armonk/NY, USA). Normal distribution was assessed using the Kolmogorov-Smirnov test. P-values below 0.05 were assumed to indicate statistical significance.

The average stenosis scores of HR-QISS and CE-MRA were compared using a nonparametric Wilcoxon signed-rank test. In addition to the segment-based analysis, per-region and per-limb analyses were performed. For these analyses, the segment grades were averaged for each of the three regions (distal aorta and pelvis, thigh, and calf) and each limb. A subgroup analysis was performed to assess the performance of HR-QISS in distinguishing between high-grade stenosis (grades 2 and 3) and occlusion (grade 4).

Sensitivity, specificity, positive predictive value (PPV), and negative predictive value (NPV) of the non-enhanced HR-QISS for the detection of significant (≥50%, grades 2–4) stenoses were assessed relative to the CE-MRA as the reference standard. For the purpose of per-region and per-limb calculations, the highest grade – indicating the most severe stenosis – was identified for the region and limb, respectively.

Diagnostic image quality values of HR-QISS and CE-MRA were compared by using the paired t-test and were reported as means ± standard deviation. The level of interreader agreement of stenosis severity assessment for both MRA techniques was assessed by Cohen’s Kappa (κ) [21].

## Results

Evaluation of QISS-MRA and CE-MRA was possible in all included patients (N = 21). 13 patients were male, 8 were female. Mean age was 72.9±7.8 years. The clinical manifestation of PAOD reached from Fontaine stage IIb to stage IV. In particular, it was stage IIb in 11 patients, stage III in 4 patients, and stage IV in 6 patients. Assuming a typical heart rate of 72 BPM (RR-interval 0.833 sec), the net imaging time for HR-QISS was 12.4 minutes. Including table movement and shimming times, the actual mean total time of HR-QISS examination was 21 minutes.

Overall, 551 of the 567 arterial segments were assessable by HR-QISS, while 555 out of 567 arterial segments were assessable by CE-MRA. Segments with a stent (8) or a vascular prosthesis (4) were non-assessable and had to be excluded in both MRA techniques. Four segments were non-assessable in HR-QISS due to signal dropout caused by endoprosthesis. For the evaluation of image quality, 42 segments with no visible artery due to total occlusion in the whole segment of the respective artery were excluded.

In total, 42 limbs, 63 regions, and 551 anatomic segments were assessed with respect to the degree of stenosis and 42 limbs, 58 regions and 513 segments with regard to image quality.

### Overall Diagnostic Performance of Non-enhanced MRA

For HR-QISS, 174 of 551 segments (31.6%) were rated as significantly stenosed (≥50%) and 377 segments (68.4%) as not significantly stenosed (<50%). A significant stenosis was diagnosed in 176 of 551 segments (31.9%) according to the rating of the CE-MRA. There was no systematic over- or underestimation of the presence of significant stenosis using non-enhanced MRA (p>0.05).

In the segment-based pairwise comparison, non-enhanced MRA showed no significant difference in the stenosis grades in any of the 14 anatomical segments (each with p>0.05). Overall, for HR-QISS, a sensitivity of 94.1%, a specificity of 97.8%, a positive predictive value (PPV) of 95.1%, and a negative predictive value (NPV) of 97.2% were obtained.

In the region-based analysis, non-enhanced and contrast-enhanced MRA techniques showed similar maximum stenosis grades in all three regions (distal aorta and pelvis, thigh, and calf; each with p>0.05). HR-QISS showed a sensitivity of 97.0%, a specificity of 96.7%, a PPV of 97.0%, and a NPV of 96.7%.

In the limb-based analysis, no difference was found in the pairwise comparison. All limbs were assigned the same maximum stenosis grade in the HR-QISS as compared to CE-MRA, resulting in limb-based sensitivity, specificity, PPV and NPV of each 100%.

**Figure 2:**
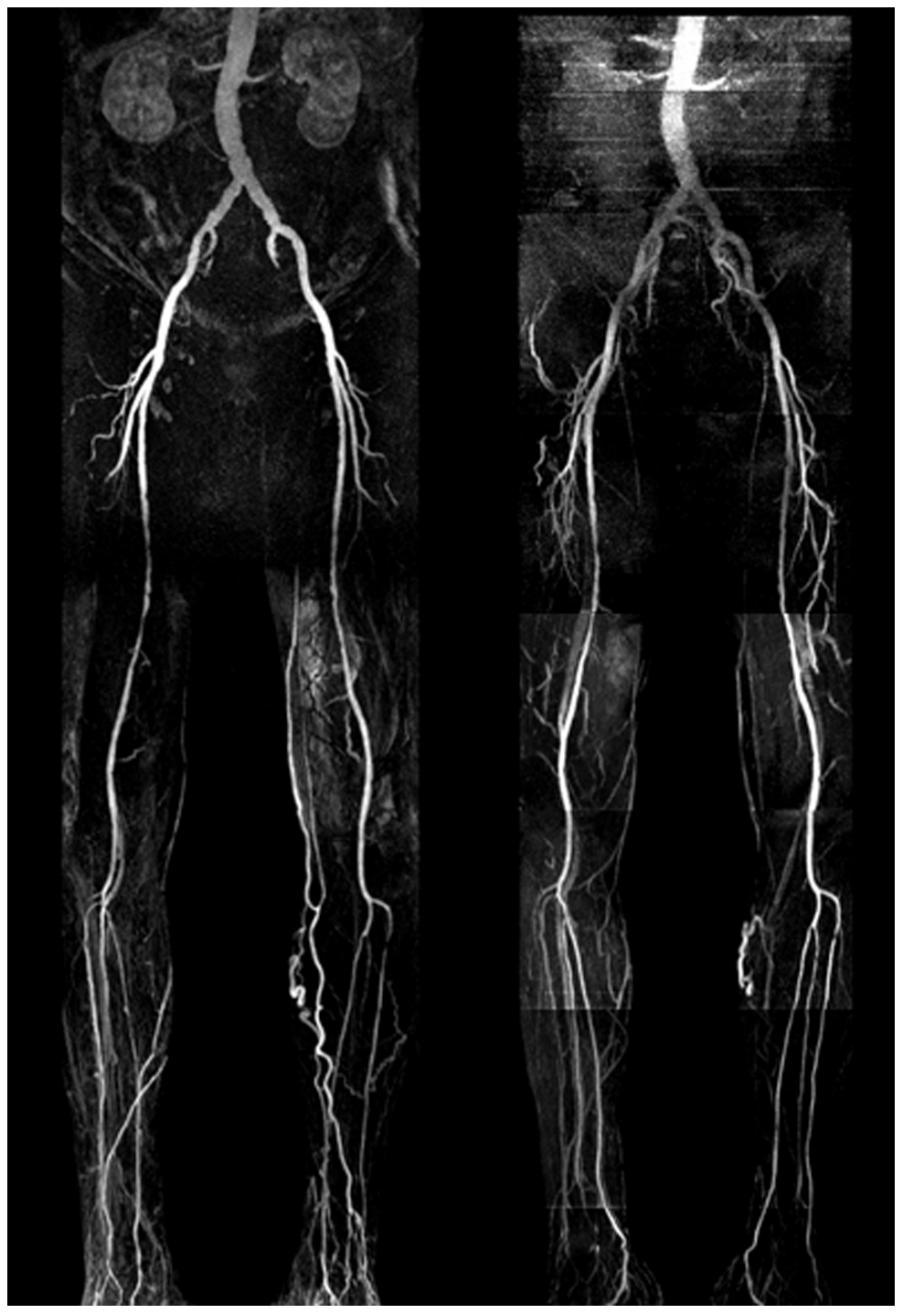
Contrast-enhanced continuous table movement (left) and non-enhanced HR-QISS (right) MRA in a 68 y/o male patient suffering from PAOD stage IV. The examination was performed after percutaneous transluminal angioplasty (PTA) of the right superficial femoral artery. Both CE-MRA and HR-QISS depict multiple significant (>50%) stenoses in the right superficial femoral artery, an occlusion of the right anterior tibial artery and a single significant short-distance stenosis in the proximal left anterior tibial artery. Note the slightly better image quality of CE-MRA in the distal aorta due to motion artifacts in the HR-QISS sequence. On the other hand, non-enhanced HR-QISS shows less venous overlay.

**Figure 3:**
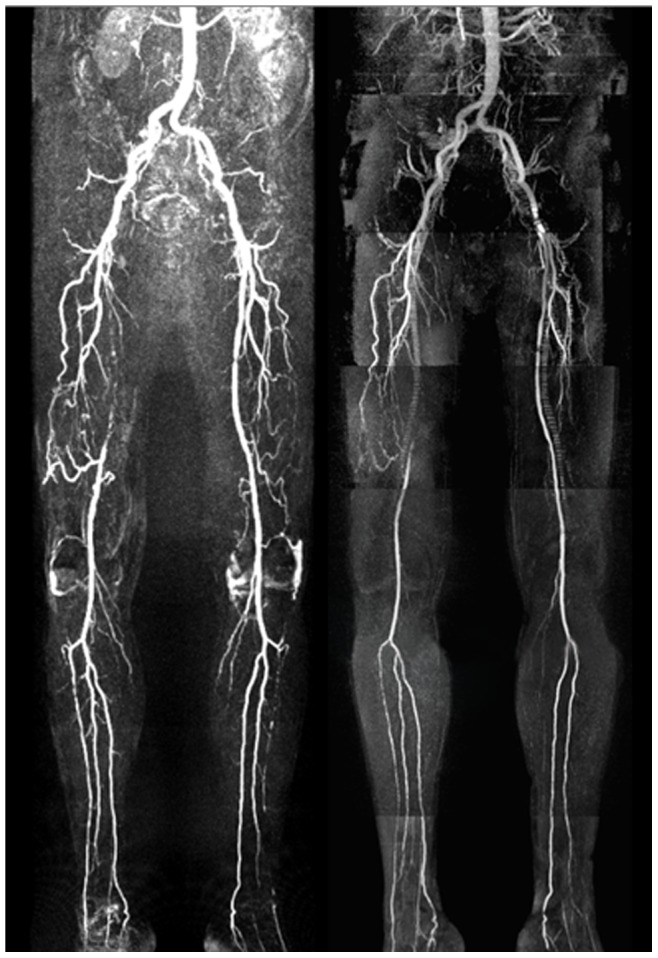
Contrast-enhanced (left) and non-enhanced HR-QISS (right) MRA in a 72 y/o male with PAOD stage IIb. CE-MRA and HR-QISS findings show excellent correlation. Both techniques reveal a long-distance occlusion of the right superficial femoral artery. The right superficial femoral artery is reconstituted via collaterals from the deep femoral artery. Further occlusions are found in both fibular arteries and the proximal left anterior tibial artery. High-grade stenoses are located in the right anterior tibial artery and in the left superficial femoral artery.

The results of the diagnostic performance of HR-QISS for the detection of stenosis in the lower extremities are summarized in Table 2. Clinical examples are shown in Figures 2, 3.

**Table 2:** Diagnostic performance of non-enhanced high-resolution QISS-MRA vs. contrast enhanced MRA, N = 21.

	Per segment	Per region	Per limb
Sensitivity (%)	94.1 (89.8–96.7)	97.0 (83.3–99.4)	100.0 (91.6–100.0)
Specificity (%)	97.8 (95.8–98.8)	96.7 (84.7–99.5)	100.0 (91.6–100.0)
PPV (%)	95.1 (91.2–98.3)	97.0	100.0
NPV (%)	97.2 (95.1–98.6)	96.7	100.0

Numbers in brackets are 95% confidence intervals. NPV: negative predictive value; PPV: positive predictive value.

### Distinguishing between High-grade Stenosis and Occlusion

In summary, 176 segments were rated as significantly stenosed or occluded (grade 2 or higher) in the CE-MRA reading. Within this subgroup, using HR-QISS, 62 segments (35.2%) were rated as highly stenosed (grades 2 and 3) and 114 segments (64.8%) were rated as occluded (grade 4). For CE-MRA, 58 segments (33.0%) were classified to have a high-grade stenosis and 118 segments (77.0%) to have an occlusion.

For the detection of an occlusion in all segments with a stenosis grade of 2 and higher in the CE-MRA reference standard, HR-QISS showed a sensitivity of 93.1%, a specificity of 93.2%, a PPV of 96.5%, and a NPV of 87.1%.

### Comparison of Diagnostic Image Quality

Subjective diagnostic image quality of the anatomical segments was rated as excellent or good in 333/513 (64.9%) segments using HR-QISS and in 393/513 (76.6%) segments using CE-MRA, respectively.

Overall, mean image quality of QISS-MRA was 2.58±0.91, while it was significantly higher in CE-MRA (2.84±1.06, p<0.01, paired t-test). Image quality of QISS-MRA was significantly lower for the regions of the distal aorta and the pelvis (p<0.01), and for the region of thigh (p<0.01). No significant difference was found in the region of the calf (p = 0.78). Table 3 shows detailed information on diagnostic image quality comparison.

**Table 3:** Per-region image quality of non-enhanced high-resolution QISS-MRA vs. contrast enhanced MRA, N = 21.

	Distal aorta and pelvis	Thigh	Calf
HR-QISS	2.44±0.90	2.80±0.70	2.52±1.10
CE-MRA	3.11±1.12	3.24±0.74	2.51±1.01
t-statistics	10.05	7.64	0.29
p-value*	<0.01	<0.01	0.78

Data are mean diagnostic image quality Likert scores ± standard deviations, depending on the investigated anatomic region.

*Paired t-test; CE-MRA: Contrast-enhanced MR angiography; QISS-MRA: Non-enhanced quiescent-interval single-shot MR angiography.

### Interreader Agreement

There was excellent interreader agreement between stenosis scores (significant vs. non-significant stenosis) for both QISS-MRA (κ = 0.951) and CE-MRA (κ = 0.962).

## Discussion

In this prospective MRA study, non-enhanced ECG-gated high-resolution QISS-MRA was successfully applied for the visualization of the peripheral arteries on a 3 Tesla MR system in patients with advanced PAOD. As compared to contrast-enhanced MRA as standard of reference, sensitivity, specificity, PPV and NPV were high. The overall image quality of HR-QISS was fair, but significantly lower in the distal aorta, the iliac and the femoral arteries when compared to CE-MRA. No significant difference was found in the more distal anatomical segments.

Our findings are consistent with and extend those of previous studies on non-enhanced MRA of the lower extremities. While several well-known non-enhanced MRA techniques such as Time-of-Flight (TOF)-MRA [22], phase contrast MRA [23], and Turbo-Spin-Echo (TSE) [18], [24], [25], have shown to be more or less inadequate for the investigation of PAOD, QISS-MRA has already shown satisfactory results in the assessment of the peripheral arteries. In comparison to CE-MRA, sensitivities ranged from 84.9% to 98.6% and specificities from 94.6% to 96.8% [11]–[13], [16].

We could show that HR-QISS with a slice thickness of 1.5 mm at 3 Tesla performs very well in the presence of advanced disease. HR-QISS apparently profits from an improved contrast between arteries and surrounding soft tissue due to a higher field strength. In our patient collective, less than 1% of segments were not assessable in HR-QISS but only in CE-MRA. Sensitivity and specificity of HR-QISS were 94.1% and 97.8%, respectively. Remarkably, in contrast to previous studies, no significant difference between HR-QISS and CE-MRA was found with regard to stenosis grade in any of the anatomical segments. Furthermore, the ability of HR-QISS to distinguish between high-grade stenosis and occlusion was very good. An overestimation of severe stenosis as it has been reported for QISS-MRA techniques at 1.5 Tesla could not be observed in our study.

In our study, we included only patients with a confirmed PAOD of Fontaine stage IIb and higher. It is important to note that previous studies had included subjects in which PAOD is suspected but not necessarily confirmed. Advanced PAOD, however, can cause major variations in blood flow velocity among different segments, making MR imaging considerably more difficult. PAOD patients also often suffer from cardiac arrhythmia and they tend to be elderly and may suffer from back pain or restless legs which can cause motion artifacts. Therefore, we excluded patients with absent or only mild symptoms of PAOD in order not to overestimate the diagnostic performance of QISS in its target patient group.

Although image quality is less crucial than diagnostic accuracy, it remains a drawback in QISS-MRA. Similarly to previous reports, image quality in the distal aorta, pelvis and thigh was significantly lower in HR-QISS. This is probably also due to the fact that we completely avoided breath holding in order to increase patient comfort.

The main advantage of HR-QISS is that no gadolinium-based contrast agent is needed, which can potentially cause nephrogenic systemic fibrosis [5], [6]. As patients suffering from PAOD have a high prevalence of chronic renal failure, HR-QISS is particularly well-suited for this patient population [10]. Moreover, non-enhanced techniques increases patient comfort, and cost for contrast agent can be avoided. Another benefit of QISS as compared to other non-enhanced techniques is that imaging parameters typically do not have to be tailored to the patient’s heart rate or other factors. In case of bulk motion, the slice group containing the region of concern can be repeated.

While HR-QISS offers many advantages, it is also important to bear in mind that total examination times of over twenty minutes can cause problems especially in elder and severely ill patients. Recent technical developments, namely a QISS technique that uses a highly undersampled radial k-space trajectory, has proven to enable the acquisition of two to three slices per cardiac cycle [26]. Thereby, it was possible to shorten the scan time for a complete peripheral MRA to 2 minutes or less.

The presence of signal dropouts due to stents and metal clips is another reason to prefer CE-MRA in certain patients. Also, even if we did not encounter any problems with ECG gating, it has been reported that low QRS voltage or poor lead contact can result in artifacts [12], [27]. ECG gating is the most popular method for cardiac synchronization; however, it is subject to interference from switching magnetic field gradients and radiofrequency pulses. In this context, a recently presented approach of a self-navigated non-enhanced QISS-MRA that does not require the use of ECG gating appears promising [28].

We acknowledge a few limitations of our study. First, correlations of MRA findings to the gold standard, invasive DSA, were not performed. This reflects the fact that DSA is mainly reserved for candidates for revascularization therapy at our institution. We therefore used CE-MRA, which is generally considered as the non-invasive gold standard [3], [4]. Second, it was not possible to completely blind the readers as to whether the MRA examination was contrast-enhanced or not because of distinguishing features such as contrast agent in the bladder cannot be hidden. Third, the data analysis was performed on 5-point Likert scales and we did not quantify artery diameters and stenosis length. However, we think that this approach reflects the reading of an MRA study as it is performed in clinical practice rather than the evaluation by using additional quantitative tools.

Finally, we included a relatively small number of patients in this study. We are convinced, however that it is sufficient to demonstrate the eligibility of HR-QISS as we obtained excellent results, although the study exclusively focused on critical patients with a poor vascular status.

In conclusion, non-enhanced HR-QISS MRA at 3 Tesla shows a high sensitivity and specificity in the evaluation of the peripheral arteries at a reasonable imaging time. HR-QISS performs very well even in patients even with advanced PAOD and is particularly well suited for patients for which the administration of contrast agent is associated with a high risk of NSF. It seems to become apparent that QISS is the most suitable technique for non-contrast-enhanced MR angiography of the lower extremities.
